# Earlier bedtime at 1.5 years is associated with greater height gain at 3 years of age: a nationwide birth cohort study of the Japan environment and children’s study

**DOI:** 10.3389/fpubh.2026.1945746

**Published:** 2026-09-24

**Authors:** Shintaro Senoo, Keita Shimizu, Masanobu Fujimoto, Yukiko Yamaguchi, Hiroshi Sunada, Toshio Masumoto, Akemi Morita, Hisashi Noma, Noriyuki Namba, on behalf of the Japan Environment Children's Study Group

**Affiliations:** 1 Division of Pediatrics & Perinatology, Faculty of Medicine, Tottori University, Yonago, Tottori, Japan; 2 Advanced Medicine, Innovation and Clinical Research Center, Tottori University Hospital, Yonago, Tottori, Japan; 3 Division of Health Administration and Promotion, Faculty of Medicine, Tottori University, Yonago, Tottori, Japan; 4 Department of Interdisciplinary Statistical Mathematics, The Institute of Statistical Mathematics, Tachikawa, Tokyo, Japan

**Keywords:** birth cohort, linear growth, sleep duration, sleep timing, toddler

## Abstract

**Background:**

Previous studies have shown inconsistent associations between sleep duration and height gain in early childhood. The relationships of bedtime and nighttime awakenings with growth remain unclear. We investigated whether sleep characteristics at 1.5 years were associated with subsequent height gain.

**Methods:**

Forty-nine thousand two hundred eight singleton, term toddlers classified as appropriate for gestational age were selected from the Japan Environment and Children’s Study. Nighttime sleep was defined as sleep between 20:00 and 07:59. Nighttime sleep duration, bedtime, and nighttime awakenings were categorized as long (≥10 h) or short (<10 h), early (20:00–21:59) or late (≥22:00), and infrequent (<3) or frequent (≥3), respectively. Linear mixed-effects models were used to examine associations with height and height standard deviation scores (SDS).

**Results:**

The early bedtime group showed greater monthly increases in height and height SDS than the late bedtime group, corresponding to 18-month differences of 0.13 cm (*B* = 7.21 × 10^−^3 cm/month, 95% confidence interval (CI) 0.06 to 0.20; *p* < 0.001) and 0.046 SD (*B* = 2.53 × 10^−^3 SD/month, 95% CI 0.027 to 0.064; *p* < 0.001), respectively. The long sleep group did not differ significantly from the short sleep group in either outcome in the primary multivariable model. In continuous analyses, sleep duration was significantly associated with height gain, but not with height SDS gain. In an additional analysis including sleep outside the predefined nighttime window, the >14-h group showed smaller increases in height and height SDS than the 11–14-h group, whereas the <11-h group did not differ significantly from the 11–14-h group. The infrequent nighttime awakenings group did not differ significantly from the frequent nighttime awakenings group in either outcome.

**Conclusion:**

Earlier bedtime at 1.5 years was associated with greater height gain, although the magnitude of the association was small. The associations of sleep duration with height gain varied between nighttime and 24-h sleep analyses. The observed association with earlier bedtime may have practical relevance because bedtime can be discussed during routine child health guidance; however, given the observational study design, these findings should not be interpreted as evidence of a causal effect.

## Introduction

Sleep is generally considered to have a positive effect on childhood growth. However, sleep is not included in the infancy-childhood-puberty (ICP) model, which is proposed as a growth pattern for children (1). Previous studies on the associations of sleep duration with height gain in early childhood have shown inconsistent results (2–5). Furthermore, studies showing no significant association between sleep duration and height have not examined other potentially relevant sleep dimensions, including sleep timing and sleep fragmentation.

Recent evidence suggests that the impact of sleep on health cannot be fully explained by its quantitative aspect alone. Factors such as sleep regularity and circadian misalignment (e.g., social jetlag) are closely associated with a wide range of health outcomes, including mortality risk and daytime functioning (6, 7). Sleep is a multidimensional behavior that encompasses several dimensions beyond sleep duration, including sleep timing and sleep fragmentation. In a literature review, sleep experts identified sleep latency, awakenings lasting more than 5 min, wake after sleep onset, and sleep efficiency—the ratio of total sleep time to time in bed—as indicators of sleep quality (8). However, these indicators of sleep quality do not encompass other dimensions of sleep, such as sleep timing and sleep fragmentation. Given that sleep is involved in key physiological processes such as endocrine regulation and tissue growth, it is plausible that different dimensions of sleep—including sleep timing and sleep fragmentation— may influence physical growth, specifically linear height gain, in early childhood. Nevertheless, no study has directly investigated the associations of these sleep dimensions with height gain in early childhood.

Kawai et al. utilized a large-scale birth cohort in the Japan Environment and Children’s Study (JECS) (9, 10) and demonstrated that nighttime sleep duration of ≥9.5 h at 1.5 years of age was associated with higher odds of tall stature (≥75th percentile among participants) at 3 years of age, compared with a sleep duration of ≤9 h (11). However, the study primarily focused on sleep duration, and other sleep dimensions, including sleep timing and sleep fragmentation, were not incorporated into the analytical model. Therefore, it remains unclear whether sleep timing and sleep fragmentation independently contribute to longitudinal height gain.

Our study aimed to investigate the relationship between sleep and height in more detail by assessing sleep duration, sleep timing, and sleep fragmentation within the same cohort. We investigated the respective associations of these sleep dimensions with longitudinal height gain during early childhood. We hypothesized that an earlier bedtime, longer nighttime sleep duration, and fewer nighttime awakenings would be associated with greater height gain during this developmental period.

## Methods

### Study population

The JECS is an ongoing nationwide birth cohort study. The JECS aims to elucidate environmental factors that influence children’s health and development. The JECS protocol was reviewed and approved by the Ministry of the Environment’s Institutional Review Board on Epidemiological Studies and the Ethics Committees of all participating institutions (No. 100910001). Pregnant women were recruited between January 2011 and March 2014 from Co-operating healthcare providers or municipal offices. Written informed consent was obtained from all participants. These data have been used in numerous studies. Details regarding the JECS are described in the literature (9, 10).

This study used the JECS dataset (jecs-ta-20190930) released in October 2019. This dataset includes 104,062 fetal records. It contains no identifying information regarding the participants or caregivers. Anthropometric parameters, including height, were primarily measured at nursery schools, municipal health centers, and medical facilities, and recorded by caregivers. Height data at 1.5, 2, 2.5, and 3 years of age were collected from questionnaire responses administered by mail. The exclusion criteria were as follows: miscarriages or stillbirths (*n* = 3,759), multiple births (*n* = 1,891), preterm or post-term births (gestational age less than 37 or greater than 42 weeks), or incomplete data (*n* = 5,132), unknown sex and birth physical data (*n* = 283). Furthermore, children with a birth weight or length < −2 SD (*n* = 2,408) or > + 2 SD (*n* = 4,246), based on the sex-specific standard data on birth weight and length stratified by gestational age in the Japanese population, and physical abnormalities (*n* = 9,908) were excluded. Among the 76,435 term infants classified as appropriate for gestational age without physical abnormalities, we excluded children for whom the year or month of the measurement date was unknown at any of the assessment points (1 month, 1.5 years, 2 years, 2.5 years, and 3 years; *n* = 26,177). This exclusion was necessary because the precise age at each follow-up point (1.5, 2, 2.5, and 3 years) is not always exactly 18, 24, 30, or 36 months; therefore, accurate fractional ages needed to be calculated using the difference between the measurement date at each specific point and that at the 1-month baseline. Furthermore, because height standard deviation scores (SDS) values at each age were rigorously calculated monthly, participants lacking year or month information of measurement were excluded. In addition, participants with missing information on nighttime sleep or those with a reported nighttime sleep duration of 0 h at evaluation were excluded as input errors (*n* = 27). Additionally, at each checkpoint of 1.5, 2, 2.5, and 3 years, height data with a height SDS < −3 SD or > + 3 SD (*n* = 26), as well as cases with one or fewer assessable height SDS values (*n* = 997), were excluded. Finally, 49,208 toddlers were included in the analysis (Figure 1).

**Figure 1:**
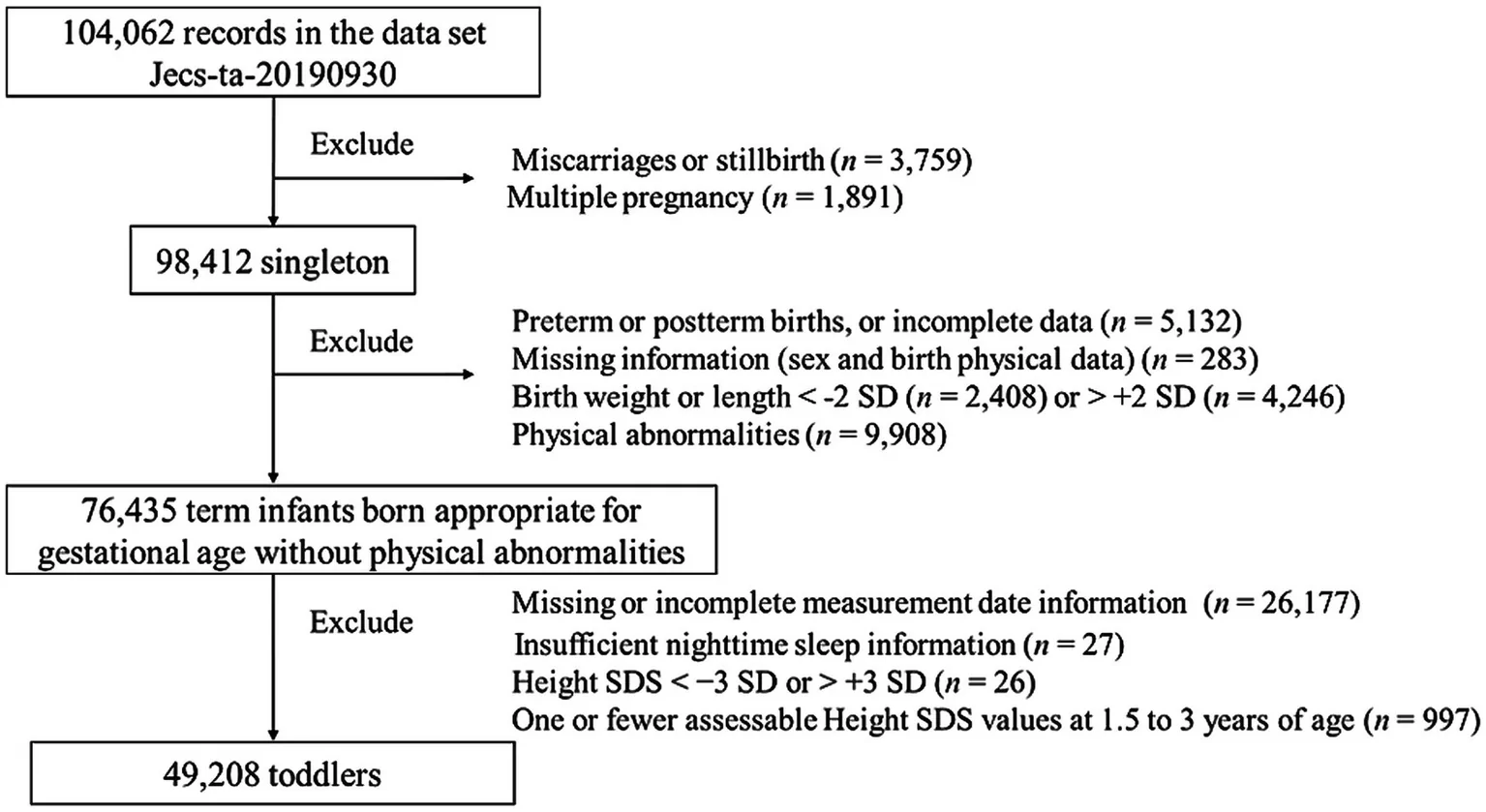
Flowchart detailing the entry of research participants. A comprehensive review of 104,062 records was conducted in accordance with the exclusion criteria. The analysis included 49,208 participants with sufficient data on anthropometric measurements and sleep data. AGA, appropriate for gestational age; SDS, Standard Deviation Score.

### Sleep assessment and definitions

Caregivers were asked to report their child’s sleep over a 24-h period, from midnight to midnight on the following day, by marking whether the child was asleep or awake at each 0.5-h interval in response to the following question: “Please report the time your child slept yesterday.” The distribution of sleep recorded across the 24-h period and the number of children with sleep recorded at each time point are shown in Supplementary Figure 1. To evaluate three distinct sleep dimensions (sleep duration, sleep timing, and sleep fragmentation), nighttime sleep was operationally defined as sleep occurring between 20:00 and 07:59 on the following day. The nighttime sleep window was established for the present study based on two considerations: the general guidance on sleep duration and healthy sleep habits for young children provided by the Ministry of Health, Labour, and Welfare of Japan (12), and the relatively late bedtimes observed among children in Japan compared with those in other countries (13). The guidance provides recommendations on the amount of sleep rather than specifying a fixed bedtime or wake time. In this study, sleep duration refers to the total sleep time within this predefined nighttime sleep period, whereas sleep timing and sleep fragmentation were assessed using bedtime and nighttime awakenings, respectively. Daytime sleep was not included in the primary sleep duration analysis because, given the 0.5-h recording intervals, it was difficult to clearly distinguish daytime naps from sleep extending continuously from the predefined nighttime sleep period. Sleep data were limited to variables available from the standardized JECS questionnaire and did not include objective measures such as actigraphy. Although the questionnaire was administered consistently across the JECS, it is not a formally validated pediatric sleep instrument.

#### Sleep duration groups

Within the predefined nighttime sleep period, sleep duration was calculated by summing the recorded sleeping intervals. The American Academy of Sleep Medicine (AASM) recommends 11–14 h of total daily sleep time, including naps, for children aged 1–2 years (14), and this recommendation is also used as a reference in Japan. Because the present study focused specifically on sleep during the predefined nighttime sleep period, rather than total 24-h sleep, we classified children with nighttime sleep duration of ≥ 10 h into the “long sleep group” and those with < 10 h into the “short sleep group.” This cutoff was selected with reference to the sleep duration recommendations of the AASM and the guidance provided by the Japanese government (12).

#### Bedtime groups

Bedtime was defined as the start time of the first sleeping interval that was followed by another consecutive 0.5-h sleeping interval, indicating at least 1 h of continuous sleep within the predefined nighttime sleep period. In defining the bedtime groups, we considered the relatively late average bedtime of 21:17 reported for children aged 0–3 years in Japan (13) and the bedtime distribution among 1-year-old children reported in a previous study using JECS data (15). In that study, approximately 65% of children went to bed after 21:00 and approximately 20% after 22:00. Accordingly, bedtime between 20:00 and 21:59 was classified as the “early bedtime group,” whereas bedtime at or after 22:00 was classified as the “late bedtime group.” Because the observation window began at 20:00, children who had fallen asleep before 20:00 were classified as belonging to the “early bedtime group.”

#### Nighttime awakening groups

Sleep fragmentation was assessed by the number of nighttime awakenings. Based on the available questionnaire data and previous reports (8, 16), each transition from a sleeping interval to an awake interval within the predefined nighttime sleep period was counted as a nighttime awakening. A previous study reported 2.5 nighttime awakenings as the upper limit for 1-year-old children (16). Accordingly, participants with < 3 nighttime awakenings were classified as the “infrequent awakenings group,” whereas those with ≥ 3 nighttime awakenings were classified as the “frequent awakenings group.”

To assess the robustness of the selected cutoff values for sleep duration, bedtime, and nighttime awakenings, we performed sensitivity analyses using alternative cutoff thresholds (Supplementary Table 1).

### Anthropometric measurements

The child’s sex, gestational age at birth, and neonatal anthropometric measurements were transcribed from childbirth medical records by Co-operating healthcare providers. Gestational age in weeks and days was determined through the first-trimester ultrasound examination and/or an estimation based on the last menstrual period. Additionally, height data at 1.5, 2, 2.5, and 3 years of age were extracted. Height SDS values at birth and each measurement point were calculated according to standard growth charts for Japanese children using the lambda-mu-sigma (LMS) method (17, 18) Parental height SDS was calculated using Japanese reference values at age 17.5 years, which approximates final adult height in the Japanese population. We acknowledge that the use of reference values at 17.5 years of age may lead to a slight underestimation of true adult height in a small proportion of individuals. However, height gain beyond late adolescence is minimal in the general population, and thus the impact on SDS estimation is expected to be negligible. The LMS values at this age were used to calculate the height SDS for parental heights. In addition, parents with a height SDS < −3 SD or > + 3 SD were excluded from the analysis to minimize the potential influence of extreme parental heights (analytical sample: 47,872 mothers and 47,196 fathers).

### Covariates

Socioeconomic information and lifestyle-related data were self-reported during early and/or mid-pregnancies (19). Parental height has been reported to influence children’s growth (20). Household income and parental education level, which are known to affect children’s sleep, were included as covariates (21). The variables were dichotomized as parents’ last education being either senior high school or higher and household income being either below or above 6 million yen per year. This classification was based on the 2010 summary report of the Comprehensive Survey of Living Conditions, which indicated that the annual income of households with children was 6.07 million yen in Japan (22). In addition, assuming that children in households with cohabiting working parents tend to have later bedtimes, the number of income earners in the same household was included as a covariate. Households with one or fewer earners were classified as single-earner households, whereas those with two or more earners were classified as dual-earner households.

### Statistical analyses

Data cleaning was performed using Python 3.8.16 with Pandas 1.5.3 (23). Statistical analyses were performed using R (R Foundation for Statistical Computing, Vienna, Austria) and EZR (Saitama Medical Center, Jichi Medical University, Saitama, Japan), a graphical user interface for R ver. 4.3.3 (24). Linear mixed-effects models (LMMs) (25) were fitted using the lmer function from the lme4 package (version 1.1–35.1). To evaluate the longitudinal associations between baseline sleep factors measured at 1.5 years and subsequent height gain (or height SDS change) between 1.5 and 3 years of age, LMMs included fixed effects for the sleep variable, time, their interaction, and the required covariates. Time was treated as a continuous variable according to age in years. A random intercept for each child was included to account for within-child correlation among repeated measurements. No random slope for time was included. By incorporating the interaction between the sleep variable and time, the coefficients quantified the difference in the monthly rate of height growth between sleep groups (cm/month or SD/month). LMMs were fitted using restricted maximum likelihood and included all available longitudinal observations for each child; thus, children with missing height measurements at individual visits were not excluded from the longitudinal analysis. Univariable and multivariable analyses were conducted using LMMs. In the primary multivariable analysis, sleep duration, bedtime, nighttime awakenings, sex, and socioeconomic factors (parental height SDS, parental education level, household income, and number of income earners) were included as explanatory variables. Sleep duration, bedtime, and nighttime awakenings were analyzed as categorical variables, and sleep duration was additionally analyzed as a continuous variable. A separate multivariable model excluding bedtime and nighttime awakenings was used to examine the association between nighttime sleep duration and growth, with sleep duration analyzed both categorically and continuously (Supplementary Table 2). An additional analysis examined the association between total 24-h sleep duration, including all sleep outside the predefined nighttime window, and growth (Supplementary Table 3), using 11–14 h, the recommended total daily sleep duration for children aged 1–2 years, as the reference category. An additional sensitivity analysis was performed to assess the potential impact of misclassification of sleep duration resulting from the predefined nighttime sleep period. In this analysis, 373 children identified as having been misclassified into the short sleep group were reclassified into the long sleep group, and the multivariable model was refitted with sleep duration analyzed as a categorical variable (Supplementary Table 4). We also performed sensitivity analyses using two alternative nighttime sleep periods, 19:30–07:59 and 20:00–08:29, to assess the influence of the predefined time boundaries on the results. For each alternative nighttime sleep period, nighttime sleep duration and the number of nighttime awakenings were recalculated according to the corresponding time window, while the definition of bedtime remained unchanged. The same multivariable model as that used in the primary analysis was then refitted using these recalculated sleep variables (Supplementary Tables 5a,b, respectively). To evaluate whether socioeconomic factors were associated with sleep characteristics, linear regression analyses were performed with sleep duration, bedtime, and nighttime awakenings as outcome variables and socioeconomic factors as explanatory variables. In the univariable regression analysis, sleep duration and nighttime awakenings were treated as continuous variables. Welch’s t-test was used to compare mean height and height SDS at 1.5 years among groups defined by sleep factors and covariates. Normality of parental height SDS was assessed using the Kolmogorov–Smirnov (K-S) test. Statistical significance was defined as a *p*-value < 0.05. Because this study analyzed an existing cohort and included all eligible participants, no *a priori* sample size calculation was performed. To characterize the statistical sensitivity of our main analyses, minimum detectable effects (MDEs) were calculated for the sleep-factor coefficients in the primary multivariable models using the model-based standard errors from the fitted linear mixed-effects models, assuming 80% power and a two-sided alpha level of 0.05. The MDE was calculated as (z_0.975_ + z_0.80_) × SE. Given the exploratory nature of the secondary comparisons across baseline characteristics and subgroups (Tables 1–4), formal adjustments for multiple testing, such as false discovery rate correction, were not applied. Therefore, the reported *p*-values for these comparisons are unadjusted and should be interpreted as exploratory findings.

**Table 1:** Sleep factors (sleep duration, bedtime, nighttime awakenings), height, and height SDS profiles at each time point.

Sleep factors (*N* = 49,208)	Height (cm)			Height SDS		
Sleep duration	Long sleep group (≥ 10 h)	Short sleep group (< 10 h)	*p*-value	Long sleep group (≥ 10 h)	Short sleep group (< 10 h)	*p*-value
1.5 years of age	77.96 ± 3.29	77.94 ± 3.23	0.407	−0.45 ± 1.00	−0.49 ± 1.01	<0.001
2 years of age	82.64 ± 3.24	82.62 ± 3.21	0.397	−0.40 ± 0.97	−0.44 ± 0.98	<0.001
2.5 years of age	87.43 ± 3.47	87.37 ± 3.42	0.106	−0.31 ± 0.98	−0.36 ± 0.98	<0.001
3 years of age	91.71 ± 3.50	91.60 ± 3.47	< 0.001	−0.21 ± 0.96	−0.26 ± 0.95	<0.001

Bedtime	Early bedtime group (20:00–21:59)	Late bedtime group (≥ 22:00)	*p*-value	Early bedtime group (20:00–21:59)	Late bedtime group (≥ 22:00)	*p*-value
1.5 years of age	77.94 ± 3.25	78.02 ± 3.34	0.024	−0.47 ± 1.00	−0.45 ± 1.03	0.193
2 years of age	82.64 ± 3.21	82.60 ± 3.30	0.354	−0.41 ± 0.97	−0.41 ± 0.99	0.864
2.5 years of age	87.41 ± 3.44	87.38 ± 3.51	0.490	−0.32 ± 0.98	−0.33 ± 0.99	0.163
3 years of age	91.69 ± 3.47	91.62 ± 3.57	0.111	−0.22 ± 0.95	−0.25 ± 0.97	0.003

Nighttime awakenings	Infrequent awakenings group (< 3)	frequent awakenings group (≥ 3)	*p*-value	Infrequent awakenings group (< 3)	Frequent awakenings group (≥ 3)	*p*-value
1.5 years of age	77.95 ± 3.27	77.84 ± 3.19	0.319	−0.46 ± 1.01	−0.50 ± 0.98	0.256
2 years of age	82.64 ± 3.23	82.53 ± 3.20	0.339	−0.41 ± 0.97	−0.44 ± 0.97	0.345
2.5 years of age	87.41 ± 3.45	87.21 ± 3.45	0.081	−0.32 ± 0.98	−0.42 ± 0.97	0.002
3 years of age	91.68 ± 3.49	91.41 ± 3.46	0.018	−0.22 ± 0.96	−0.32 ± 0.95	0.003

Data are presented as mean ± standard deviation (SD). The sample sizes (n) for each group at each time point varied slightly due to missing data. The sample sizes were substantially imbalanced between the nighttime awakening groups; therefore, borderline *p*-values should be interpreted with caution. The numbers of participants with available height data at 1.5, 2, 2.5, and 3 years of age, respectively, were as follows: Sleep Duration: Long sleep group, *n* = 29,626/30,708/30,543/31,039; Short sleep group, *n* = 15,923/16,416/16,351/16,586. Bedtime: Early bedtime group, *n* = 37,025/38,300/38,123/38,718; Late bedtime group, *n* = 8,524/8,824/8,771/8,907. Nighttime awakenings: Infrequent awakenings group, *n* = 44,662/46,223/45,985/46,711; Frequent awakenings group, *n* = 887/901 / 909/914.

**Table 2:** Univariable linear mixed-effects model estimates of monthly changes in height and height SDS from 1.5 to 3 years of age according to sleep characteristics, demographic characteristics, and socioeconomic factors.

Univariable model	Height *B* (×10^−3^ cm/month)	*p*-value	Height SDS *B* (×10^−3^ SD/month)	*p*-value
Sleep duration (≥ 10 h vs. < 10 h)	5.10 (2.28, 7.92)	<0.001	0.41 (−0.32, 1.13)	0.274
Sleep duration (hrs)	3.86 (2.39, 5.34)	<0.001	0.43 (0.05, 0.81)	0.026
Bedtime (20:00–21:59 vs. ≥ 22:00)	7.57 (4.12, 11.02)	<0.001	2.48 (1.59, 3.37)	<0.001
Nighttime awakenings (< 3 vs. ≥ 3)	8.31 (−1.47, 18.09)	0.096	3.01 (0.48, 5.53)	0.020
Sex (male vs. female)	−13.10 (−15.74, −10.37)	<0.001	−3.56 (−4.25, −2.86)	<0.001
Maternal height SDS	17.16 (15.80, 18.53)	<0.001	2.29 (1.94, 2.65)	<0.001
Paternal height SDS	17.00 (15.60, 18.40)	<0.001	2.49 (2.13, 2.85)	<0.001
Maternal education status (post-secondary education vs. ≤ senior high school)	5.99 (3.08, 8.89)	<0.001	3.56 (2.81, 4.31)	<0.001
Paternal education status (post-secondary education vs. ≤ senior high school)	1.87 (−0.88, 4.62)	0.183	1.67 (0.95, 2.38)	<0.001
Annual household income (≥ 6 million yen vs. < 6 million yen)	2.64 (−0.40, 5.69)	0.089	2.26 (1.47, 3.04)	<0.001
Worker (multi-earner households vs. single-earner households)	3.28 (0.49, 6.06)	0.021	3.92 (3.20, 4.64)	<0.001

B, regression coefficient; CI, confidence interval; SDS, standard deviation score. Values are presented as B (95% CI). Estimates represent the monthly change in height or height SDS. For categorical variables, estimates represent the difference between the indicated group and the reference group, with comparisons shown as [target group] vs. [reference group]. For continuous variables, estimates represent the change per unit increase in the variable.

**Table 3:** Multivariable linear mixed-effects model estimates of monthly changes in height and height SDS from 1.5 to 3 years of age according to sleep characteristics, with adjustment for demographic and socioeconomic factors.

Multivariable model	Height *B* (×10^−3^ cm/month)			*p*-value
	Categorical sleep duration (≥ 10 h vs. < 10 h)	*p*-value	Continuous sleep duration (per hour)	
Sleep duration	1.45 (−1.82, 4.71)	0.385	2.47 (0.69, 4.25)	0.006
Bedtime (20:00–21:59 vs. ≥ 22:00)	7.21 (3.26, 11.15)	<0.001	5.40 (1.34, 9.46)	0.009
Nighttime awakenings (< 3 vs. ≥ 3)	6.49 (−3.97, 16.95)	0.222	3.50 (−7.14, 14.14)	0.519
Sex (male vs. female)	−13.29 (−16.11, −10.46)	<0.001	−13.22 (−16.04, −10.39)	< 0.001
Maternal height SDS	16.38 (14.94, 17.81)	<0.001	16.37 (14.93, 17.80)	< 0.001
Paternal height SDS	16.27 (14.81, 17.72)	<0.001	16.25 (14.80, 17.71)	< 0.001
Maternal education status (post-secondary education vs. ≤ senior high school)	3.32 (0.03, 6.60)	0.048	3.32 (0.03, 6.60)	0.048
Paternal education status (post-secondary education vs. ≤ senior high school)	−1.01 (−4.09, 2.07)	0.520	−1.06 (−4.14, 2.01)	0.499
Annual household income (≥ 6 million yen vs. < 6 million yen)	−2.39 (−5.68, 0.89)	0.153	−2.30 (−5.59, 0.98)	0.169
Worker (multi-earner households vs. single-earner households)	3.01 (0.00, 6.01)	0.049	3.33 (0.32, 6.34)	0.030

Height SDS *B* (×10^−3^ SD/month)			
Categorical sleep duration (≥ 10 h vs. < 10 h)	*p*-value	Continuous sleep duration (per hour)	*p*-value
−0.34 (−1.19, 0.51)	0.436	0.02 (−0.44, 0.48)	0.944
2.53 (1.51, 3.56)	<0.001	2.36 (1.31, 3.42)	<0.001
2.62 (−0.09, 5.34)	0.058	2.39 (−0.38, 5.16)	0.091
−3.60 (−4.34, −2.87)	<0.001	−3.60 (−4.33, −2.86)	<0.001
2.17 (1.79, 2.54)	<0.001	2.17 (1.79, 2.54)	<0.001
2.36 (1.98, 2.74)	<0.001	2.36 (1.98, 2.73)	<0.001
2.41 (1.56, 3.27)	<0.001	2.41 (1.56, 3.27)	<0.001
0.53 (− 0.27, 1.33)	0.191	0.53 (−0.27, 1.33)	0.193
0.13 (−0.72, 0.99)	0.760	0.14 (−0.71, 1.00)	0.744
3.72 (2.94, 4.54)	<0.001	3.75 (2.97, 4.50)	<0.001

B, regression coefficient; CI, confidence interval; SDS, standard deviation score. Data are presented as estimates (B) with 95% confidence intervals (CIs) in parentheses. Estimates represent the monthly change in height or height SDS. The multivariable model was simultaneously adjusted for all variables listed in the table, including sleep duration, bedtime, nighttime awakenings, sex, maternal height SDS, paternal height SDS, maternal education status, paternal education status, annual household income, and number of household earners. For categorical variables, estimates represent the difference between the indicated group and the reference group, with comparisons shown as [target group] vs. [reference group]. For continuous variables, estimates represent the change per unit increase in the variable.

**Table 4:** Linear regression analysis of the associations between socioeconomic factors and sleep characteristics at 1.5 years of age

Socioeconomic factor	Outcome	*B*	*SE*	*p*-value	Adj. *R^2^*
Maternal education status	Post-secondary education vs. ≤ senior high school				
	Sleep duration	0.03	<0.001	<0.001	<0.001
	Bedtime	−0.01	<0.01	<0.001	<0.01
	Nighttime awakenings	0.06	<0.01	<0.001	<0.01
Paternal education status	Post-secondary education vs. ≤ senior high school				
	Sleep duration	0.04	<0.01	<0.001	<0.01
	Bedtime	−0.07	<0.01	<0.001	<0.01
	Nighttime awakenings	0.03	<0.01	<0.001	<0.01
Annual household income	≥ 6 million yen vs. < 6 million yen				
	Sleep duration	−0.06	< 0.01	< 0.001	< 0.01
	Bedtime	−0.06	< 0.01	< 0.001	< 0.01
	Nighttime awakenings	0.08	< 0.01	< 0.001	< 0.01
Worker	Multi-earner households vs. single-earner households				
	Sleep duration	−0.15	< 0.01	< 0.001	< 0.01
	Bedtime	0.00	< 0.01	0.91	< 0.01
	Nighttime awakenings	0.10	< 0.01	< 0.001	< 0.01

B, regression coefficient; SE, standard error. B represents the difference in the indicated sleep factor between the target and reference groups. The target and reference groups are shown in the “Socioeconomic factor” column. Adj. *R*^2^ represents the adjusted coefficient of determination for each regression model. *p*-values < 0.05 were considered statistically significant.

## Results

The demographic characteristics, including anthropometric characteristics, sleep characteristics, and socioeconomic factors, of the 49,208 study participants and their caregivers are presented in Table 5. The gestational age was 39.47 ± 1.11 weeks, and 50.1% of the children were male. Height increased from 49.1 ± 1.7 cm at birth to 91.7 ± 3.5 cm at 3 years of age, while height SDS changed from 0.04 ± 0.83 to −0.23 ± 0.96. Parental height SDS was 0.01 ± 0.99 for maternal and 0.17 ± 0.97 for paternal. At 1.5 years of age, 65.2% of children were in the long sleep group (≥ 10 h), 81.2% in the early bedtime group (20:00–21:59), and 98.1% in the infrequent nighttime awakenings group. Regarding socioeconomic factors, 68.1% of maternal and 59.3% of paternal had post-secondary education, 28.6% of households had an annual income of ≥ 6 million yen, and 60.3% were multi-earner households. The K–S test indicated statistically significant departures from normality for parental height (*p* < 2.2 × 10^−16^), whereas skewness and kurtosis were minimal, suggesting that the distributions of maternal and paternal height closely approximated normality.

**Table 5:** Anthropometric characteristics, sleep characteristics, and socioeconomic factors of the analyzed participants and their caregivers.

Anthropometric data	Valid *N* (Male: %)	Height (cm)	Height SDS	Weight (kg)
at birth	49,208 (50.1)	49.1 ± 1.7	0.04 ± 0.83	3.05 ± 0.32
1.5 years of age	45,549 (50.1)	78.0 ± 3.3	−0.46 ± 1.01	10.11 ± 1.08
2 years of age	47,124 (50.2)	82.6 ± 3.2	−0.41 ± 0.97	11.21 ± 1.21
2.5 years of age	46,894 (50.1)	87.4 ± 3.5	−0.32 ± 0.98	12.43 ± 1.33
3 years of age	47,625 (50.1)	91.7 ± 3.5	−0.23 ± 0.96	13.46 ± 1.42
Maternal height	47,872	158.2 ± 5.3	0.01 ± 0.99	–
Paternal height	47,196	171.8 ± 5.6	0.17 ± 0.97	–

Sleep characteristics	Categories	Valid *N*	Values, *n* (%)
Sleep duration	Long sleep group (≥ 10 h)	49,208	32,101 (65.2)
	Short sleep group (< 10 h)		17,107 (34.8)
Bedtime	Early bedtime group (20:00–21:59)	49,208	39,968 (81.2)
	Late bedtime group (≥ 22:00)		9,240 (18.8)
Nighttime awakenings	Infrequent awakenings group (< 3)	49,208	48,251 (98.1)
	Frequent awakenings group (≥ 3)		957 (1.9)

Socioeconomic factors	Categories	Valid *N*	Values, *n* (%)	Missing, *n* (% of total)
Maternal education status	Post-secondary education	48,847	33,264 (68.1)	361 (0.7)
Paternal education status	Post-secondary education	48,637	28,850 (59.3)	571 (1.2)
Annual household income	≥ 6 million yen	46,231	13,245 (28.6)	2,977 (6.0)
Earners	Multi-earner households	47,679	28,768 (60.3)	1,529 (3.1)

Data are presented as mean ± standard deviation (SD) for continuous variables and as *n* (%) for categorical variables. Valid *N* represents the number of participants with non-missing data for each variable. Percentages are calculated based on Valid *N*. Missing data for socioeconomic factors are presented as *n* (%) of the analyzed participants.

Table 1 shows the distributions of sleep duration, bedtime, and nighttime awakenings in relation to height and height SDS at each time point. Among the 49,208 participants, the number of participants with available height data varied across follow-up time points due to missing data. The long sleep group was larger than the short sleep group, whereas the frequent awakenings group was particularly small. At 1.5 years of age, the baseline time point for the LMM analysis, mean height SDS was significantly higher in the long sleep group than in the short sleep group (*p* < 0.001). In contrast, mean height was significantly higher in the late bedtime group than in the early bedtime group (*p* = 0.024). Over follow-up, significant differences in height between the long and short sleep groups emerged at 3 years of age (*p* < 0.001), while significant differences between nighttime awakening groups emerged at 2.5 and 3 years of age.

To investigate the associations of participants’ sleep characteristics and background factors with their growth trajectories, univariable and multivariable analyses were conducted. The results of the univariable analysis are presented in Table 2. When evaluated as categorical groups, the long sleep group exhibited greater monthly increases in height than the short sleep group (*B* = 5.10 × 10^−3^ cm/month, *p* < 0.001), whereas the difference in height SDS was not statistically significant (*B* = 0.41 × 10^−3^ SD/month, *p* = 0.274). The early bedtime group showed significantly greater monthly increases in both height (*B* = 7.57 × 10^−3^ cm/month, *p* < 0.001) and height SDS (*B* = 2.48 × 10^−3^ SD/month, *p* < 0.001). For nighttime awakenings, the infrequent awakenings group showed a significantly greater monthly increase in height SDS (*B* = 3.01 × 10^−3^ SD/month, 95% CI 0.48 × 10^−3^ to 5.53 × 10^−3^, *p* = 0.020), whereas the difference in height was not statistically significant (*B* = 8.31 × 10^−3^ cm/month, *p* = 0.096). When evaluated as a continuous variable, longer sleep duration (per hour) was significantly associated with greater monthly increases in both height (*B* = 3.86 × 10^−3^ cm/month, 95% CI 2.39 × 10^−3^ to 5.34 × 10^−3^, *p* < 0.001) and height SDS (*B* = 0.43 × 10^−3^ SD/month, 95% CI 0.05 × 10^−3^ to 0.81 × 10^−3^, *p* = 0.026). Female sex, parental height SDS, maternal education status, and multi-earner household status were significantly associated with greater monthly increases in both height and height SDS. Paternal education status and annual household income were significantly associated with height SDS, but not height. Sensitivity analyses using alternative categorical cutoffs in univariable models for sleep duration, bedtime, and nighttime awakenings yielded generally consistent associations for bedtime, whereas the associations for sleep duration and nighttime awakenings varied depending on the cutoff used (Supplementary Table 1).

Multivariable analysis (Table 3) demonstrated greater monthly increases in both height and height SDS in the early bedtime group than in the late bedtime group (height: *B* = 7.21 × 10^−3^ cm/month, 95% CI 3.26 × 10^−3^ to 11.15 × 10^−3^, *p* < 0.001; height SDS: *B* = 2.53 × 10^−3^ SD/month, 95% CI 1.51 × 10^−3^ to 3.56 × 10^−3^, *p* < 0.001). Regarding sleep duration, when evaluated as a continuous variable in the primary multivariable model that also included bedtime and nighttime awakenings, sleep duration remained significantly associated with height in centimeters (*B* = 2.47 × 10^−3^ cm/month, 95% CI 0.69 × 10^−3^ to 4.25 × 10^−3^, *p* = 0.006), whereas its association with height SDS was not statistically significant (*B* = 0.02 × 10^−3^ SD/month, 95% CI − 0.44 × 10^−3^ to 0.48 × 10^−3^, *p* = 0.944). When evaluated as a categorical variable, sleep duration was not significantly associated with either height or height SDS in the primary multivariable model (*p* = 0.385 and *p* = 0.436, respectively). In a separate multivariable model excluding bedtime and nighttime awakenings, continuous sleep duration was significantly associated with both height (*B* = 3.57 × 10^−3^ cm/month, 95% CI 2.02 × 10^−3^ to 5.13 × 10^−3^, *p* < 0.001) and height SDS (*B* = 0.53 × 10^−3^ SD/month, 95% CI 0.13 × 10^−3^ to 0.93 × 10^−3^, *p* = 0.010; Supplementary Table 2). In an additional analysis of total 24-h sleep duration, including all sleep outside the predefined nighttime window (Supplementary Table 3), the >14-h group (*n* = 2,800) showed significantly smaller monthly increases in both height (*B* = −7.48 × 10^−3^ cm/month, 95% CI − 13.66 × 10^−3^ to −1.29 × 10^−3^, *p* = 0.018) and height SDS (*B* = −2.79 × 10^−3^ SD/month, 95% CI − 4.40 × 10^−3^ to −1.19 × 10^−3^, *p* < 0.001) than the 11–14-h group (*n* = 39,652), whereas no significant differences were observed in the < 11-h group (*n* = 6,756; height, *p* = 0.121; height SDS, *p* = 0.961). In the models presented in Table 3, male sex was negatively associated with monthly increases in both height and height SDS, whereas maternal and paternal height SDS were positively associated with both outcomes. Higher maternal education and multi-earner household status were positively associated with monthly increases in both height and height SDS, whereas paternal education and annual household income were not significantly associated with either outcome.

For the primary model in which sleep duration, bedtime, and nighttime awakenings were evaluated, the approximate MDEs over the 18-month follow-up period were 0.082 cm for sleep duration, 0.101 cm for bedtime, and 0.380 cm for nighttime awakenings for the height outcome; the corresponding values for height SDS were 0.027, 0.026, and 0.088 SD, respectively. The frequent awakenings group included 957 participants, compared with 48,251 in the infrequent awakenings group. The adjusted estimate for the frequent versus infrequent awakenings groups corresponded to an 18-month difference of approximately 0.117 cm (95% CI − 0.071 to 0.305 cm) in height and 0.047 SD (95% CI − 0.002 to 0.096 SD) in height SDS.

The results of the analyses of socioeconomic factors potentially associated with sleep factors are presented in Table 4. Linear regression analyses showed that sleep duration was longer in groups with higher maternal and paternal education levels, whereas it was shorter in groups with higher annual household income and in multi-earner households (compared with single-earner households). Bedtime was earlier in groups with higher maternal and paternal education levels and higher annual household income, whereas no statistically significant association was observed with household work status (*p* = 0.91). The number of nighttime awakenings was higher in groups with higher maternal and paternal education levels, higher annual household income, and multi-earner households.

## Discussion

Through LMM analysis using the large dataset from the JECS, a nationwide birth cohort study, we found that earlier bedtime was significantly associated with greater height gain from 1.5 to 3 years of age, whereas the association between sleep duration and height gain varied according to how sleep duration was modeled and which other sleep characteristics were included in the model. More notably, the early bedtime group showed significantly greater monthly increases in both height and height SDS than the late bedtime group, with differences of 7.21 × 10^−3^ cm/month and 2.53 × 10^−3^ SD/month, respectively, in the multivariable analysis. These estimates correspond to approximately 0.13 cm and 0.046 SD greater gains, respectively, over the 18-month follow-up period. Although the mean height of the early bedtime group was significantly lower at 1.5 years, this difference was no longer statistically significant at 3 years (*p* = 0.111). While the LMM demonstrated significant positive associations between earlier bedtime and greater height gain, regression to the mean cannot be completely excluded and should therefore be considered when interpreting these findings. In contrast, the number of nighttime awakenings was not significantly associated with height gain. The absence of a statistically significant association between nighttime awakenings and height gain should be interpreted with caution. The frequent awakenings group included only 957 children, compared with 48,251 in the infrequent awakenings group, resulting in substantially lower statistical power and precision than for the comparisons of sleep duration and bedtime. The 95% CI remained compatible with modest differences in subsequent growth (e.g., an 18-month difference of approximately 0.117 cm [95% CI − 0.071 to 0.305 cm] for height and 0.047 SD [95% CI − 0.002 to 0.096 SD] for height SDS), and the approximate MDEs over the 18-month follow-up period were approximately 0.380 cm for height and 0.088 SD for height SDS. Thus, the absence of a statistically significant association does not rule out a small association between nighttime awakenings and subsequent growth. Sensitivity analyses using alternative categorizations of sleep duration, bedtime, and the number of nighttime awakenings were also conducted (Supplementary Table 1). The association with earlier bedtime was generally consistent across alternative categorizations, whereas the associations of sleep duration and nighttime awakenings varied depending on the cutoff used.

In contrast, the association between bedtime and growth may reflect the timing and alignment of sleep with growth-related endocrine and circadian rhythms. Growth hormone (GH) secretion is closely linked to sleep onset and slow-wave sleep, with the major nocturnal GH pulse generally occurring shortly after sleep onset in association with deep or slow-wave sleep (26, 27). Thus, earlier sleep onset may be more closely aligned with the timing of this major GH pulse. In addition, in young children, later bedtimes are associated with delayed melatonin onset, and evening light exposure can delay circadian phase (28), suggesting that earlier bedtime may reflect better alignment between sleep and the endogenous circadian rhythm. However, because sleep stages, GH secretion, melatonin, and light exposure were not assessed in the present study, these mechanisms remain speculative.

Consistent with a previous study (20), height gain was influenced by parental height. In addition, maternal education status and multi-earner household status were positively associated with monthly increases in both height and height SDS in the multivariable analysis. These findings indicate that family socioeconomic characteristics were associated with differences in early childhood growth, although these associations should not be interpreted as causal relationships.

Previous studies revealed inconsistent findings on the association between sleep duration and height with various sample sizes and ethnicities (2–5). Kawai et al. reported an association between height and sleep duration using multivariable analyses in the JECS dataset (11). In contrast, our analysis showed that when bedtime and the number of nighttime awakenings were included in the LMM, sleep duration remained significantly associated only with height in centimeters when modeled continuously (*B* = 2.47 × 10^−3^ cm/month, *p* = 0.006), whereas its association with height SDS was no longer statistically significant (*p* = 0.944; Table 3). When sleep duration was evaluated as a categorical variable, it was not significantly associated with either height or height SDS. However, when bedtime and the number of nighttime awakenings were excluded from the model, continuous sleep duration was significantly associated with both height (*B* = 3.57 × 10^−3^ cm/month, *p* < 0.001) and height SDS (*B* = 0.53 × 10^−3^ SD/month, *p* = 0.010; Supplementary Table 2). These findings indicate that the association between continuous sleep duration and longitudinal height gain was attenuated after adjustment for bedtime and nighttime awakenings, particularly for height SDS. This difference from the findings of Kawai et al. may reflect differences in the sleep variables included in the models. A strength of the present study is that we evaluated three distinct sleep dimensions—sleep duration, bedtime, and nighttime awakenings—and considered them simultaneously in the multivariable analysis. Although both studies were based on the same large JECS cohort, they addressed different research questions and therefore complement each other. Together, they provide more comprehensive evidence regarding the association between sleep and longitudinal height gain in early childhood.

To further examine the association between sleep duration and growth when total 24-h sleep duration, including all sleep outside the predefined nighttime window, was considered, we performed an additional analysis using an 11–14-h reference group (Supplementary Table 3). Compared with the 11–14-h group, children with a total sleep duration of > 14 h showed significantly smaller monthly increases in both height (*B* = −7.48 × 10^−3^ cm/month, *p* = 0.018) and height SDS (*B* = −2.79 × 10^−3^ SD/month, *p* < 0.001), whereas no significant differences were observed in the <11-h group. These findings differed from the pattern observed in the primary analysis based on nighttime sleep duration, indicating that the observed associations varied according to the definition and categorization of sleep duration. As this additional analysis was exploratory, these findings should be interpreted cautiously and do not establish a causal association between total sleep duration and subsequent growth.

Several limitations should be acknowledged. First, although sleep information was collected at multiple time points in the JECS using the same questionnaire-based approach, our analysis used only the assessment at 1.5 years of age. We selected sleep at 1.5 years as the exposure because we hypothesized that sleep characteristics at this age, rather than those at 3 years, may be more relevant to subsequent changes in physical growth through 3 years of age. Nevertheless, sleep schedules, including bedtime, can change substantially during early childhood, and a single assessment at 1.5 years may not fully represent sleep characteristics throughout the follow-up period and may have resulted in exposure misclassification.

As shown in Supplementary Figure 1, the number of children who were asleep increased around 20:00 (19:30: *n* = 2,524 [5.1%]; 20:00: *n* = 6,361 [12.9%]; 20:30: *n* = 14,675 [29.8%]) and decreased around 08:00 (07:30: *n* = 13,525 [27.5%]; 08:00: *n* = 6,289 [12.8%]; 08:30: *n* = 3,424 [7.0%]). This distribution indicates that the transition into and out of sleep occurred predominantly around the boundaries of the predefined nighttime period. These findings support the appropriateness of the 20:00–07:59 definition for capturing the typical nighttime sleep period in this study population. However, the use of this predefined nighttime period of 20:00–07:59 may have resulted in some misclassification of sleep duration. To assess the potential impact of this definition, we examined representative sleep patterns in which children had a continuous sleep episode without any recorded awakening that extended beyond either boundary of the predefined nighttime sleep period. For example, children who fell asleep before the 20:00 boundary (such as at 19:00 or 19:30) had their sleep duration truncated at 20:00, meaning that their sleep duration within the predefined window was undercounted and could potentially result in misclassification into the short sleep group. Similarly, children who remained asleep beyond the 07:59 boundary (such as until 08:59 or 09:29) had their sleep duration truncated at 07:59, which could likewise result in underestimation of sleep duration and misclassification. These patterns included sleep extending before the 20:00 boundary (19:00–05:29, *n* = 66; 19:00–05:59, *n* = 105; 19:30–05:59, *n* = 112) and sleep extending beyond the 07:59 boundary (22:00–08:29, *n* = 38; 22:00–08:59, *n* = 31; 22:30–08:59, *n* = 10; 22:30–09:29, *n* = 11). In total, 373 children (0.76%) were identified as having these specific boundary-crossing sleep patterns that could potentially result in misclassification of sleep duration. Importantly, these 373 children represent only the specific patterns examined and should not be interpreted as the total number of potentially misclassified children, because the available 30-min interval data did not allow us to systematically distinguish sleep outside the predefined nighttime period from separate sleep episodes, such as evening naps before 20:00 or morning naps after 08:00. In a sensitivity analysis, these 373 children were reclassified from the short sleep group to the long sleep group based on their identifiable continuous sleep episodes. The results of the multivariable model treating sleep duration as a categorical variable were largely consistent with those of the primary analysis (Supplementary Table 4). The same definition also limited the precision with which bedtime could be assessed. For children who fell asleep before 20:00, the exact bedtime was not further specified, as any bedtime before 20:00 would have been classified into the early bedtime group under the predefined operational definition and therefore would not have affected the primary analysis of the association between bedtime category and subsequent height gain.

To further assess the potential influence of the predefined nighttime sleep period, we performed sensitivity analyses using two alternative nighttime sleep periods, 19:30–07:59 and 20:00–08:29 (Supplementary Tables 5a,b, respectively). In these analyses, nighttime sleep duration and nighttime awakenings were recalculated according to the respective time window, while the bedtime definition remained unchanged. The results were largely consistent with those of the primary analysis (Table 3). In particular, the positive association between continuous sleep duration and monthly height gain remained statistically significant using both alternative definitions (19:30–07:59: *B* = 2.61 × 10^−3^ cm/month, *p* = 0.004; 20:00–08:29: *B* = 1.89 × 10^−3^ cm/month, *p* = 0.019), compared with *B* = 2.47 × 10^−3^ cm/month (*p* = 0.006) in the primary analysis. The association between earlier bedtime and greater monthly height gain was also highly consistent across the three definitions (primary analysis: *B* = 7.21 × 10^−3^ cm/month, *p* < 0.001; 19:30–07:59: *B* = 7.30 × 10^−3^ cm/month, *p* < 0.001; 20:00–08:29: *B* = 7.08 × 10^−3^ cm/month, *p* < 0.001). These findings suggest that, although some degree of misclassification related to the predefined nighttime sleep period cannot be excluded, changing the boundaries of the nighttime sleep period did not materially alter the observed associations.

Relatedly, daytime naps could not be clearly defined and separately evaluated in the primary analysis. Because the present study aimed to examine sleep duration, bedtime, and nighttime awakenings as distinct sleep characteristics, we first needed to define a consistent nighttime sleep period. Sleep occurring during the non-nighttime period (08:00–19:59) could not always be clearly classified as a daytime nap or as part of a sleep episode extending across the predefined nighttime period, particularly given that sleep was recorded in 30-min intervals. Therefore, rather than attempting to define daytime naps using complex case-specific rules, we focused the primary analysis on the predefined nighttime sleep period. As a complementary analysis, we additionally examined total 24-h sleep duration, including all sleep outside the predefined nighttime window, in relation to subsequent height gain (Supplementary Table 3). However, this analysis could not distinguish the independent contributions of nighttime sleep and sleep occurring outside the predefined nighttime window.

In addition, residual confounding by underlying health conditions cannot be excluded, particularly for the > 14-h sleep group. Children with physical abnormalities identified by 1 month of age were excluded from the study population. Information on several health conditions reported at approximately 1 year of age could be obtained from the questionnaire, including asthma, atopic dermatitis, food allergy, neonatal/infantile gastrointestinal allergy, Kawasaki disease, allergic rhinitis, pollinosis, cerebral palsy, psychomotor developmental delay, diabetes mellitus, leukemia, hematologic malignancies, brain tumors, and neuroblastoma, as well as free-text responses. However, these conditions were not systematically used as exclusion criteria or covariates because it was difficult to reliably identify chronic conditions that could be associated with both prolonged sleep and impaired growth, given the heterogeneity of the conditions and limited clinical detail available from the questionnaire. Thus, residual confounding cannot be ruled out, and reverse causality is also possible, as underlying health conditions affecting growth may contribute to longer sleep duration. These limitations should be considered when interpreting the findings for total 24-h sleep duration.

Second, a substantial number of participants were excluded because information necessary for calculating accurate age at each anthropometric assessment was unavailable. Specifically, children for whom the year or month of the measurement date was unknown at one or more assessment points were excluded (*n* = 26,177), because precise fractional ages could not be calculated and height SDS at each assessment point could not be accurately determined. An additional 27 children were excluded because of missing information on nighttime sleep or a reported nighttime sleep duration of 0 h, which was considered an input error. Although these exclusions were based on predefined data-quality criteria, the exclusion of a substantial number of participants may have introduced selection bias and may have limited the generalizability of our findings.

Third, sleep characteristics were based on caregiver-reported questionnaire data and therefore may have been subject to recall or reporting error. Caregiver reports were used because continuous monitoring of children’s sleep throughout the night is difficult in a large-scale cohort study. Although sleep information was collected using a standardized questionnaire, objective methods such as actigraphy or polysomnography were not available to verify sleep duration, bedtime, or nighttime awakenings. Future studies using objective sleep measures are warranted. In contrast, anthropometric measurements in the JECS were conducted by trained medical professionals or research staff according to standardized procedures designed to minimize variability across measurement settings and personnel. A strength of the present study is its comprehensive evaluation of these distinct sleep dimensions in relation to longitudinal growth.

Fourth, nutritional factors that play an important role in early childhood growth were not fully accounted for because detailed information on dietary intake, such as caloric and protein intake and feeding schedules, was not available for the present analysis. Therefore, residual confounding by nutritional status cannot be excluded. Sleep and dietary behaviors may also be interrelated and jointly influenced by circadian and metabolic processes, with the timing of sleep and food intake potentially affecting metabolic and physiological functions (29). Future studies incorporating detailed assessments of both dietary intake and the timing of food consumption alongside sleep characteristics are warranted to further elucidate the interplay between sleep, nutrition, and early childhood growth.

Fifth, daytime behaviors that may influence both bedtime and growth-related routines, including physical activity, sedentary behavior, and evening screen exposure, were not assessed in the present study. These behaviors are interrelated across the 24-h day and may influence sleep timing and other health-related behaviors in children; therefore, residual confounding by unmeasured daytime behaviors cannot be excluded (30).

Finally, some of the reference standards and contextual thresholds used in this study were established before the JECS cohort period, including the 2000 national growth survey for height SDS and the 2010 Comprehensive Survey of Living Conditions for household income. Although these standards provided consistent criteria for the analyses, secular changes in growth patterns, household income, and family routines may have occurred between the reference periods and the JECS study period; therefore, these reference values and thresholds may not fully reflect the characteristics of the study generation.

This longitudinal analysis of a large-scale birth cohort in the JECS revealed that earlier bedtime at 1.5 years of age was associated with greater height gain from 1.5 to 3 years of age, although the magnitude of the association was small. The associations of sleep duration with height gain varied between nighttime and 24-h sleep analyses. Given that Japanese infants and toddlers tend to have later bedtimes than those reported in many other countries (13), the observed association with earlier bedtime may have practical relevance because bedtime can be readily discussed as part of routine child health guidance. However, given the observational study design, these findings should not be interpreted as evidence of a causal effect, and further studies are needed to determine whether earlier bedtime causally contributes to greater height gain.

## Data Availability

The data analyzed in this study is subject to the following licenses/restrictions: data are unsuitable for public deposition due to ethical restrictions and legal framework of Japan. It is prohibited by the Act on the Protection of Personal Information (Act No. 57 of 30 May 2003, amendment on 9 September 2015) to publicly deposit the data containing personal information. Ethical Guidelines for Medical and Health Research Involving Human Subjects enforced by the Japan Ministry of Education, Culture, Sports, Science and Technology and the Ministry of Health, Labour and Welfare also restricts the open sharing of the epidemiologic data. All inquiries about access to data should be sent to: jecs-en@nies.go.jp. The person responsible for handling enquiries sent to this e-mail address is Shoji F. Nakayama, JECS Programme Office, National Institute for Environmental Studies.
