# Supplementary material for: Earlier bedtime at 1.5 years is associated with greater height gain at 3 years of age: a nationwide birth cohort study of the Japan environment and children’s study
Source: Front Public Health. 2026 Sep 24;14:1945746. doi: 10.3389/fpubh.2026.1945746 (PMC13646113; doi:10.3389/fpubh.2026.1945746)
Supplement: Supplementary file 1 [file Supplementary_file_1.DOCX]

**Supplementary Tables 1 to 5 and Supplementary Figure 1 for:**

**Earlier Bedtime at 1.5 Years is Associated with Greater Height Gain at 3 Years of Age: A Nationwide Birth Cohort Study of the Japan Environment and Children's Study**

**Contents:**

p.2	Supplementary Table 1. Sensitivity analyses of the associations between sleep characteristics and height and height SDS using univariable linear mixed-effects models

p.3-4	Supplementary Table 2. Multivariable linear mixed-effects model estimates of monthly changes in height and height SDS from 1.5 to 3 years of age according to sleep characteristics, with adjustment for demographic and socioeconomic factors

p.5	Supplementary Table 3. Multivariable linear mixed-effects model estimates of monthly changes in height and height SDS according to total sleep duration, including all sleep outside the predefined nighttime window

p.6	Supplementary Table 4. Sensitivity analysis of the associations between sleep characteristics and monthly increases in height and height SDS after reclassification of potentially misclassified children

p.7-8	Supplementary Table 5a. Multivariable linear mixed-effects model estimates of monthly changes in height and height SDS from 1.5 to 3 years of age according to sleep characteristics using an alternative nighttime sleep period of 19:30–07:59

p.9-10	Supplementary Table 5b. Multivariable linear mixed-effects model estimates of monthly changes in height and height SDS from 1.5 to 3 years of age according to sleep characteristics using an alternative nighttime sleep period of 20:00–08:29

p.11	Supplementary Figure 1. Distribution of sleep recorded across the 24-hour period and the number of children with sleep recorded at each 0.5-hour time point.

# Tables

Supplementary Table 1. Sensitivity analyses of the associations between sleep characteristics and height and height SDS using univariable linear mixed-effects models

| Univariable model | Height *B*  (×10^-3^ cm/month) | *p*-value | Height SDS *B*  (×10^-3^ SD/month) | *p*-value |
| --- | --- | --- | --- | --- |
| Long sleep group |  |  |  |  |
| ≧ 8 hrs (*n* = 48,394) | 12.33 (1.73, 22.94) | 0.023 | 4.33 (1.59, 7.07) | 0.002 |
| ≧ 9 hrs (*n* = 45,901) | 9.69 (4.32, 15.05) | < 0.001 | 2.66 (1.28, 4.05) | < 0.001 |
| ≧ 10 hrs (*n* = 32,101) | 5.10 (2.28, 7.92) | < 0.001 | 0.41 (-0.32, 1.13) | 0.268 |
| ≧ 11 hrs (*n* = 8,231) | 3.43 (-0.18, 7.05) | 0.063 | -0.95 (-1.89, -0.02) | 0.046 |
| Early sleep group |  |  |  |  |
| 20:00-20:59 (*n* = 14,848) | 5.77 (2.83, 8.70) | < 0.001 | 0.55 (0.00, 1.31) | 0.100 |
| 20:00-21:59 (*n* = 39,968) | 7.57 (4.12, 11.02) | < 0.001 | 2.48 (1.59, 3.37) | < 0.001 |
| 20:00-22:59 (*n* = 47,573) | 13.81 (6.25, 21.4) | < 0.001 | 5.85 (3.90, 7.80) | < 0.001 |
| 20:00-23:59 (*n* = 48,645) | 14.9 (2.20, 27.7) | 0.022 | 7.71 (4.42, 11.00) | < 0.001 |
| Infrequent awakenings group |  |  |  |  |
| < 1 (*n* = 12,179) | -1.36 (-4.49, 1.76) | 0.394 | -2.59 (-3.40, -1.78) | < 0.001 |
| < 2 (*n* = 46,334) | 7.61 (1.88, 13.36) | 0.009 | 2.33 (0.84, 3.81) | < 0.001 |
| < 3 (*n* = 48,251) | 8.31 (-1.47, 18.09) | 0.096 | 3.01 (0.48, 5.53) | 0.019 |

The data were analyzed using univariable linear mixed-effects models. Sleep duration, bedtime, and nighttime awakenings were analyzed separately using alternative cutoff values, with the corresponding complementary groups as reference categories. The values show estimates of fixed effects with 95% confidence intervals.

| Multivariable model | Height *B* (×10^-3^ cm/month) | | |  |
| --- | --- | --- | --- | --- |
|  | Categorical sleep duration  (≥ 10 hrs vs. < 10 hrs) | *p*-value | Continuous sleep duration (per hour) | *p*-value |
| Sleep duration | 3.98 (1.01, 6.95) | 0.009 | 3.57 (2.02, 5.13) | < 0.001 |
| Sex (male vs. female) | -13.18 (-16.01, -10.36) | < 0.001 | -13.13 (-15.96, -10.31) | < 0.001 |
| Mother’s height SDS | 16.39 (14.95, 17.82) | < 0.001 | 16.67 (14.93, 17.81) | < 0.001 |
| Father’s height SDS | 16.28 (14.82, 17.74) | < 0.001 | 16.27 (14.81, 17.72) | < 0.001 |
| Maternal education status (post-secondary education vs. ≤ senior high school) | 3.57 (0.29, 6.84) | 0.033 | 3.49 (0.21, 6.78) | 0.037 |
| Paternal education status (post-secondary education vs. ≤ senior high school) | -0.92 (-4.00, 2.16) | 0.557 | -1.01 (-4.09, 2.06) | 0.519 |
| Annual household income (≥ 6 million yen vs. < 6 million yen) | -2.25 (-5.53, 1.03) | 0.179 | -2.19 (-5.47, 1.10) | 0.192 |
| Worker (multi-earner households vs. single-earner households) | 3.38 (0.38, 6.38) | 0.027 | 3.63 (0.63, 6.63) | 0.018 |

Supplementary Table 2. Multivariable linear mixed-effects model estimates of monthly changes in height and height SDS from 1.5 to 3 years of age according to sleep characteristics, with adjustment for demographic and socioeconomic factors

The data were analyzed using a linear mixed-effects model. Sleep duration was analyzed separately as a categorical variable (≥ 10 hours vs. < 10 hours) and as a continuous variable (hours). The models were adjusted for sex, maternal and paternal height SDS, maternal and paternal education status, annual household income, and household work status. Bedtime and nighttime awakenings were not included in these models. The values show estimates of fixed effects with 95% confidence intervals.

Supplementary Table 2. Multivariable linear mixed-effects model estimates of monthly changes in height and height SDS from 1.5 to 3 years of age according to sleep characteristics, with adjustment for demographic and socioeconomic factors (continued)

| Height SDS *B* (×10^-3^ SD/month) | | | |
| --- | --- | --- | --- |
| Categorical sleep duration  (≥ 10 hrs vs. < 10 hrs) | *p*-value | Continuous sleep duration (per hour) | *p*-value |
| 0.57 (-0.20, 1.34) | 0.147 | 0.53 (0.13, 0.93) | 0.010 |
| -3.57 (-4.30, -2.83) | < 0.001 | -3.56 (-4.29, -2.82) | < 0.001 |
| 2.17 (1.80, 2.54) | < 0.001 | 2.17 (1.79, 2.54) | < 0.001 |
| 2.36 (2.00, 2.74) | < 0.001 | 2.36 (1.98, 2.74) | < 0.001 |
| 2.50 (1.64, 3.35) | < 0.001 | 2.49 (1.63, 3.34) | < 0.001 |
| 0.56 (-0.24, 1.36) | 0.167 | 0.55 (-0.25, 1.35) | 0.178 |
| 0.18 (-0.67, 1.03) | 0.676 | 0.19 (-0.66, 1.04) | 0.659 |
| 3.86 (3.08, 4.64) | < 0.001 | 3.90 (3.11, 4.57) | < 0.001 |

The data were analyzed using a linear mixed-effects model. Sleep duration was analyzed separately as a categorical variable (≥ 10 hours vs. < 10 hours) and as a continuous variable (hours). The models were adjusted for sex, maternal and paternal height SDS, maternal and paternal education status, annual household income, and household work status. Bedtime and nighttime awakenings were not included in these models. The values show estimates of fixed effects with 95% confidence intervals.

Supplementary Table 3. Multivariable linear mixed-effects model estimates of monthly changes in height and height SDS according to total sleep duration, including all sleep outside the predefined nighttime window

|  | Height *B*  (×10^-3^ cm/month) | *p*-value | Height SDS *B*  (×10^-3^ SD/month) | *p*-value |
| --- | --- | --- | --- | --- |
| Total sleep duration |  |  |  |  |
| 11–14 hours (reference; *n* = 39,652) | Reference | - | Reference | - |
| < 11 hours (*n* = 6,756) | −3.29 (−7.45, 0.86) | 0.121 | −0.03 (−1.11, 1.05) | 0.961 |
| > 14 hours (*n* = 2,800) | −7.48 (−13.66, −1.29) | 0.018 | −2.79 (−4.40, −1.19) | < 0.001 |
| Sex (male vs. female) | -13.26 (-16.08, -10.43) | < 0.001 | -3.57 (-4.31, -2.84) | < 0.001 |
| Maternal height SDS | 16.39 (14.95, 17.82) | < 0.001 | 2.17 (1.80, 2.54) | < 0.001 |
| Paternal height SDS | 16.32 (14.86, 17.78) | < 0.001 | 2.37 (1.99, 2.75) | < 0.001 |
| Maternal education status (post-secondary education vs. ≤ senior high school) | 3.59 (0.31, 6.87) | 0.032 | 2.49 (1.64, 3.34) | < 0.001 |
| Paternal education status (post-secondary education vs. ≤ senior high school) | -0.95 (-4.03, 2.13) | 0.546 | 0.56 (-0.24, 1.36) | 0.170 |
| Annual household income (≥ 6 million yen vs. < 6 million yen) | -2.35 (-5.63, 0.94) | 0.161 | 0.16 (-0.69, 1.01) | 0.716 |
| Worker (multi-earner households vs. single-earner households) | 3.15 (0.15, 6.15) | 0.040 | 3.78 (3.00, 4.56) | < 0.001 |

The data were analyzed using a linear mixed-effects model. Total sleep duration, including all sleep outside the predefined nighttime window, was categorized as < 11 hours, 11–14 hours, and > 14 hours, with 11–14 hours as the reference category. The model included an interaction term between total sleep duration and time to estimate differences in monthly changes in height and height SDS between sleep-duration groups and was adjusted for sex, maternal and paternal height SDS, maternal and paternal education status, annual household income, and household work status. The values represent differences in monthly changes between each sleep-duration group and the reference group, expressed as estimates of the time-by-sleep-duration interaction terms with 95% confidence intervals.

Supplementary Table 4. Sensitivity analysis of the associations between sleep characteristics and monthly increases in height and height SDS after reclassification of potentially misclassified children

| Multivariable model | Height *B*  (×10^-3^ cm/month) | *p*-value | Height SDS *B*  (×10^-3^ SD/month) | *p*-value |
| --- | --- | --- | --- | --- |
| Sleep duration  (≥ 10 hrs vs. < 10 hrs) | 1.45 (-1.82, 4.71) | 0.386 | -0.34 (-1.19, 0.51) | 0.436 |
| Bedtime  (20:00–21:59 vs. ≥ 22:00) | 7.21 (3.26, 11.15) | < 0.001 | 2.53 (1.51, 3.56) | < 0.001 |
| Nighttime awakenings  (< 3 vs. ≥ 3) | 6.49 (-3.97, 16.95) | 0.224 | 2.62 (-0.09, 5.34) | 0.058 |
| Sex (male vs. female) | -13.29 (-16.11, -10.46) | < 0.001 | -3.60 (-4.34, -2.87) | < 0.001 |
| Maternal height SDS | 16.38 (14.94, 17.81) | < 0.001 | 2.17 (1.79, 2.54) | < 0.001 |
| Paternal height SDS | 16.27 (14.81, 17.72) | < 0.001 | 2.36 (1.98, 2.74) | < 0.001 |
| Maternal education status (post-secondary education vs. ≤ senior high school) | 3.32 (0.03, 6.60) | 0.048 | 2.41 (1.56, 3.27) | < 0.001 |
| Paternal education status (post-secondary education vs. ≤ senior high school) | -1.01 (-4.09, 2.07) | 0.520 | 0.53 (- 0.27, 1.33) | 0.191 |
| Annual household income (≥ 6 million yen vs. < 6 million yen) | -2.39 (-5.68, 0.89) | 0.153 | 0.13 (-0.72, 0.99) | 0.760 |
| Worker (multi-earner households vs. single-earner households) | 3.01 (0.00, 6.02) | 0.049 | 3.72 (2.94, 4.50) | < 0.001 |

Values are presented as *B* coefficients (95% confidence intervals). The sensitivity analysis reclassified 373 children (0.76%) who were identified as potentially misclassified into the short sleep group because of the predefined nighttime sleep period (20:00–07:59) into the long sleep group. The data were analyzed using a linear mixed-effects model. Sleep duration was analyzed as a categorical variable (≥ 10 hours vs. < 10 hours), with < 10 hours as the reference category. The model included interaction terms between time and each sleep characteristic (sleep duration, bedtime, and nighttime awakenings) to estimate differences in monthly changes in height and height SDS between the respective sleep groups and was adjusted for sex, maternal and paternal height SDS, maternal and paternal education status, annual household income, and number of income earners. The values represent differences in monthly changes between the indicated category and the reference category, expressed as estimates of the corresponding time-by-sleep-characteristic interaction terms with 95% confidence intervals.
Supplementary Table 5a. Multivariable linear mixed-effects model estimates of monthly changes in height and height SDS from 1.5 to 3 years of age according to sleep characteristics using an alternative nighttime sleep period of 19:30–07:59

| Multivariable model | Height *B* (×10^-3^ cm/month) | | |  |
| --- | --- | --- | --- | --- |
|  | Categorical sleep duration  (≥ 10 hrs vs. < 10 hrs) | *p*-value | Continuous sleep duration (per hour) | *p*-value |
| Sleep duration | 1.24 (-2.04, 4.52) | 0.457 | 2.61 (0.85, 4.36) | 0.004 |
| Bedtime  (20:00–21:59 vs. ≥ 22:00) | 7.30 (3.35, 11.25) | < 0.001 | 5.22 (1.16, 9.28) | 0.012 |
| Nighttime awakenings  (< 3 vs. ≥ 3) | 6.63 (-0.39, 17.09) | 0.214 | 3.35 (-7.28, 14.00) | 0.537 |
| Sex (male vs. female) | -13.29 (-16.12, -10.47) | < 0.001 | -13.22 (-16.04, -10.39) | < 0.001 |
| Maternal height SDS | 16.38 (14.94, 17.81) | < 0.001 | 16.36 (14.93, 17.80) | < 0.001 |
| Paternal height SDS | 16.27 (14.81, 17.72) | < 0.001 | 16.25 (14.79, 17.71) | < 0.001 |
| Maternal education status (post-secondary education vs. ≤ senior high school) | 3.32 (0.03, 6.60) | 0.048 | 3.32 (0.03, 6.60) | 0.048 |
| Paternal education status (post-secondary education vs. ≤ senior high school) | -1.01 (-4.09, 2.07) | 0.520 | -1.07 (-4.15, 2.00) | 0.495 |
| Annual household income (≥ 6 million yen vs. < 6 million yen) | -2.40 (-5.68, 0.89) | 0.152 | -2.30 (-5.58, 0.99) | 0.171 |
| Worker (multi-earner households vs. single-earner households) | 3.00 (0.00, 6.01) | 0.051 | 3.38 (0.36, 6.39) | 0.028 |

*B*, regression coefficient; CI, confidence interval; SDS, standard deviation score.
Data are presented as estimates (*B*) with 95% confidence intervals (CIs) in parentheses. The numbers of participants were as follows: sleep duration, ≥ 10 h (*n* = 32,379) and < 10 h (*n* = 16,829); bedtime, 20:00–21:59 (*n* = 39,968) and ≥ 22:00 (*n* = 9,240); nighttime awakenings, < 3 (*n* = 48,251) and ≥ 3 (*n* = 957). In this sensitivity analysis, nighttime sleep duration and nighttime awakenings were recalculated using an alternative nighttime sleep period of 19:30–07:59, while the bedtime definition remained unchanged. The multivariable model was adjusted for all variables listed in the table. For categorical variables, estimates represent differences from the reference group; for continuous variables, estimates represent changes per unit increase.

Supplementary Table 5a. Multivariable linear mixed-effects model estimates of monthly changes in height and height SDS from 1.5 to 3 years of age according to sleep characteristics using an alternative nighttime sleep period of 19:30–07:59 (continued)

| Height SDS *B* (×10^-3^ SD/month) | | | |
| --- | --- | --- | --- |
| Categorical sleep duration  (≥ 10 hrs vs. < 10 hrs) | *p*-value | Continuous sleep duration (per hour) | *p*-value |
| -0.45 (-1.30, 0.40) | 0.300 | 0.03 (-0.49, 0.43) | 0.901 |
| 2.59 (1.56, 3.61) | < 0.001 | 2.35 (1.29, 3.40) | < 0.001 |
| 2.69 (-0.03, 5.41) | 0.053 | 2.37 (-0.39, 5.13) | 0.093 |
| -3.60 (-4.34, -2.87) | < 0.001 | -3.60 (-4.33, -2.86) | < 0.001 |
| 2.17 (1.79, 2.54) | < 0.001 | 2.17 (1.79, 2.54) | < 0.001 |
| 2.36 (1.98, 2.74) | < 0.001 | 2.36 (1.98, 2.73) | < 0.001 |
| 2.41 (1.56, 3.27) | < 0.001 | 2.41 (1.56, 3.27) | < 0.001 |
| 0.53 (- 0.27, 1.33) | 0.191 | 0.53 (-0.27, 1.33) | 0.193 |
| 0.13 (-0.72, 0.98) | 0.766 | 0.14 (-0.71, 1.00) | 0.743 |
| 3.71 (2.93, 4.49) | < 0.001 | 3.76 (2.97, 4.54) | < 0.001 |

*B*, regression coefficient; CI, confidence interval; SDS, standard deviation score.
Data are presented as estimates (*B*) with 95% confidence intervals (CIs) in parentheses. The numbers of participants were as follows: sleep duration, ≥ 10 h (*n* = 32,379) and < 10 h (*n* = 16,829); bedtime, 20:00–21:59 (*n* = 39,968) and ≥ 22:00 (*n* = 9,240); nighttime awakenings, < 3 (*n* = 48,251) and ≥ 3 (*n* = 957). In this sensitivity analysis, nighttime sleep duration and nighttime awakenings were recalculated using an alternative nighttime sleep period of 19:30–07:59, while the bedtime definition remained unchanged. The multivariable model was adjusted for all variables listed in the table. For categorical variables, estimates represent differences from the reference group; for continuous variables, estimates represent changes per unit increase.

Supplementary Table 5b. Multivariable linear mixed-effects model estimates of monthly changes in height and height SDS from 1.5 to 3 years of age according to sleep characteristics using an alternative nighttime sleep period of 20:00–08:29

| Multivariable model | Height *B* (×10^-3^ cm/month) | | |  |
| --- | --- | --- | --- | --- |
|  | Categorical sleep duration  (≥ 10 hrs vs. < 10 hrs) | *p*-value | Continuous sleep duration (per hour) | *p*-value |
| Sleep duration | 2.43 (-0.78, 5.64) | 0.137 | 1.89 (0.32, 3.47) | 0.019 |
| Bedtime  (20:00–21:59 vs. ≥ 22:00) | 7.08 (3.29, 10.87) | < 0.001 | 6.30 (2.44, 10.17) | 0.001 |
| Nighttime awakenings  (< 3 vs. ≥ 3) | 5.88 (-3.90, 15.65) | 0.239 | 4.45 (-5.44, 14.33) | 0.378 |
| Sex (male vs. female) | -13.26 (-16.09, -10.43) | < 0.001 | -13.22 (-16.05, -10.39) | < 0.001 |
| Maternal height SDS | 16.38 (14.94, 17.81) | < 0.001 | 16.37 (14.93, 17.81) | < 0.001 |
| Paternal height SDS | 16.26 (14.80, 17.72) | < 0.001 | 16.26 (14.80, 17.72) | < 0.001 |
| Maternal education status (post-secondary education vs. ≤ senior high school) | 3.33 (0.03, 6.61) | 0.047 | 3.34 (0.06, 6.62) | 0.046 |
| Paternal education status (post-secondary education vs. ≤ senior high school) | -1.02 (-4.09, 2.06) | 0.517 | -1.06 (-4.13, 2.02) | 0.501 |
| Annual household income (≥ 6 million yen vs. < 6 million yen) | -2.36 (-5.64, 0.92) | 0.159 | -2.30 (-5.59, 0.99) | 0.170 |
| Worker (multi-earner households vs. single-earner households) | 3.11 (0.10, 6.12) | 0.043 | 3.33 (0.31, 6.35) | 0.031 |

*B*, regression coefficient; CI, confidence interval; SDS, standard deviation score.
Data are presented as estimates (*B*) with 95% confidence intervals (CIs) in parentheses. The numbers of participants were as follows: sleep duration, ≥ 10 h (*n* = 33,583) and < 10 h (*n* = 15,625); bedtime, 20:00–21:59 (*n* = 39,968) and ≥ 22:00 (*n* = 9,240); nighttime awakenings, < 3 (*n* = 48,113) and ≥ 3 (*n* = 1,095). In this sensitivity analysis, nighttime sleep duration and nighttime awakenings were recalculated using an alternative nighttime sleep period of 20:00–08:29, while the bedtime definition remained unchanged. The multivariable model was adjusted for all variables listed in the table. For categorical variables, estimates represent differences from the reference group; for continuous variables, estimates represent changes per unit increase.

Supplementary Table 5b. Multivariable linear mixed-effects model estimates of monthly changes in height and height SDS from 1.5 to 3 years of age according to sleep characteristics using an alternative nighttime sleep period of 20:00–08:29 (continued)

| Height SDS *B* (×10^-3^ SD/month) | | | |
| --- | --- | --- | --- |
| Categorical sleep duration  (≥ 10 hrs vs. < 10 hrs) | *p*-value | Continuous sleep duration (per hour) | *p*-value |
| -0.33 (-1.16, 0.51) | 0.441 | -0.18 (-0.59, 0.23) | 0.383 |
| 2.48 (1.49, 3.46) | < 0.001 | 2.52 (1.52, 3.53) | < 0.001 |
| 2.07 (-0.48, 4.61) | 0.111 | 2.15 (-0.42, 4.72) | 0.101 |
| -3.61 (-4.34, -2.87) | < 0.001 | -3.61 (-4.34, -2.87) | < 0.001 |
| 2.17 (1.79, 2.54) | < 0.001 | 2.17 (1.79, 2.54) | < 0.001 |
| 2.36 (1.98, 2.74) | < 0.001 | 2.36 (1.98, 2.74) | < 0.001 |
| 2.41 (1.56, 3.26) | < 0.001 | 2.41 (1.56, 3.26) | < 0.001 |
| 0.53 (- 0.27, 1.33) | 0.192 | 0.53 (-0.26, 1.34) | 0.190 |
| 0.13 (-0.72, 0.98) | 0.764 | 0.13 (-0.73, 0.98) | 0.769 |
| 3.72 (2.94, 4.51) | < 0.001 | 3.71 (2.92, 4.49) | < 0.001 |

*B*, regression coefficient; CI, confidence interval; SDS, standard deviation score.
Data are presented as estimates (*B*) with 95% confidence intervals (CIs) in parentheses. The numbers of participants were as follows: sleep duration, ≥ 10 h (*n* = 33,583) and < 10 h (*n* = 15,625); bedtime, 20:00–21:59 (*n* = 39,968) and ≥ 22:00 (*n* = 9,240); nighttime awakenings, < 3 (*n* = 48,113) and ≥ 3 (*n* = 1,095). In this sensitivity analysis, nighttime sleep duration and nighttime awakenings were recalculated using an alternative nighttime sleep period of 20:00–08:29, while the bedtime definition remained unchanged. The multivariable model was adjusted for all variables listed in the table. For categorical variables, estimates represent differences from the reference group; for continuous variables, estimates represent changes per unit increase.

# Figure legend

Supplementary Figure 1. Distribution of sleep recorded across the 24-hour period and the number of children with sleep recorded at each 0.5-hour time point.

Caregivers reported their child’s sleep status at 0.5-hour intervals over a 24-hour period, from midnight to midnight on the following day. The figure illustrates the distribution of sleep recorded across the 24-hour period and the number of children with sleep recorded at each time point.
